# Adufab‐TAT Conjugate: A Novel Approach for Transferrin Receptor‐Mediated BBB Transport in Alzheimer's Disease

**DOI:** 10.1002/alz70859_107662

**Published:** 2025-12-25

**Authors:** Moaz Elsayed Sayed Ahmed

**Affiliations:** ^1^ University of Hertfordshire, cairo, KY Egypt

## Abstract

**Background:**

This abstract aims to evaluate the enhancement of the penetration of monoclonal antibodies (mABs), specifically the aducanumab fragment (Adufab), through the blood‐brain barrier (BBB) via the transferrin receptor (TfR) by conjugating it with the trans‐activating transcriptional (TAT) peptide. This conjugation is done by the linker Glycine‐Glycine‐Glycine‐Glycine‐Serine (GGGGS)₄. This conjugation is expected to improve the selectivity and efficiency of adufab towards amyloid‐beta.

**Method:**

The structure of Adufab conjugated to TAT peptide binds to the transferrin receptor, was predicted using AlphaFold. The flexible linker (GGGGS)₄ was used to link Adufab and TAT, ensuring stable conjugation. Five models were generated, with Model 5 selected as the most reliable due to its highest pLDDT score of 64.9, indicating reliable folding, and a pTM score of 0.386, validating accurate domain arrangement. ChimeraX tools, including Axes/Planes/Centroids, Check Waters, Clashes, Contacts, Find Cavities, and H‐Bonds Render/Select by Attribute, were used to analyze the structure's stability and interactions. This in silico study focused on one‐step conjugation of Adufab to TAT and its binding to the transferrin receptor. Model 5 was identified as the best for further analysis.

**Result:**

The structural analysis of the Adufab‐TAT‐(GGGGS)₄ complex binding to the transferrin receptor revealed 367 hydrogen bonds and 4,692 non‐covalent contacts, indicating strong and specific interactions, crucial for the complex's stability and effective binding. Four steric clashes were observed, suggesting minimal structural strain. Eighteen cavities were identified, including cavities (ID 1.3.12 and ID 1.3.13) with significant volumes and depths, which could facilitate BBB transcytosis. The B‐factors ranged from 17.5 to 98.8, indicating regions of rigidity and flexibility important for the complex's ability to adapt to the dynamic BBB environment. These findings demonstrate the high binding affinity of the Adufab‐TAT conjugate for the transferrin receptor and its potential for BBB crossing. However, further experimental validation, including binding assays and biodistribution analysis, is necessary to fully evaluate its therapeutic potential.

**Conclusion:**

The Adufab‐TAT‐(GGGGS)₄ conjugate shows strong binding to the transferrin receptor, with potential for enhanced blood‐brain barrier penetration. This strategy could improve Alzheimer’s disease treatment. Future research should validate its effectiveness through experimental assays and in vivo models to confirm its therapeutic potential.

